# Paneth cell proteins DEFA6 and GUCA2A as tissue markers in necrotizing enterocolitis

**DOI:** 10.1007/s00431-023-04907-3

**Published:** 2023-04-05

**Authors:** Alice Hoffsten, Helene Engstrand Lilja, Hamid Mobini-Far, Richard Sindelar, Laszlo Markasz

**Affiliations:** 1 Department of Women’s and Children’s Health, Uppsala University, Uppsala, SE-751 85 Sweden; 2 Section of Pediatric Surgery, University Children’s Hospital, Uppsala, Sweden; 3 Department of Pathology, Uppsala University Hospital, Uppsala, Sweden; 4 Department of Surgical Sciences, Uppsala University, Uppsala, Sweden; 5 Neonatal Intensive Care Unit, University Children’s Hospital, Uppsala, Sweden

**Keywords:** Necrotizing enterocolitis, Paneth cell, Biomarker

## Abstract

Previous studies suggest that Paneth cells are involved in NEC development. Defensin alpha 6 (DEFA6) and guanylate cyclase activator 2A (GUCA2A) are selective protein markers of Paneth cells. The objective was to explore DEFA6 and GUCA2A expression in intestinal tissue samples from newborn infants with and without NEC. Tissue samples from histologically intact intestine were analyzed from 70 infants: 43 underwent bowel resection due to NEC and 27 controls were operated due to conditions such as intestinal atresia, dysmotility, aganglionosis, pseudo-obstruction or volvulus. Each tissue sample was immunohistochemically stained for DEFA6 and GUCA2A. Semi-automated digital image analysis was performed to determine protein expression. Clinical data and protein expressions were compared between the groups. DEFA6 expression was lower in the NEC group (*p* = 0.006). Low DEFA6 correlated with risk of developing NEC in a logistic regression analysis, independently of gestational age and birth weight (OR 0.843 [CI 0.732–0.971]; *p* = 0.018). GUCA2A expression did not differ between the two groups.

*Conclusion*: Lower expression of DEFA6 together with intact GUCA2A expression indicates that NEC patients have well-defined Paneth cells but diminished defensin activity. Our results suggest that DEFA6 could be used as a biomarker for NEC.

**Table:** 

**What is Known:** • *Previous studies of defensin activity in NEC have been inconsistent, showing that defensin levels may be increased or diminished in NEC. GUCA2A has to our knowledge never been studied in NEC.*	
**What is New:** • *This study benchmarks two specific Paneth cell markers (DEFA6 and GUCA2A) and their activity in individuals with and without NEC.* • *The key finding is that the NEC group had a lower DEFA6 expression compared to the Controls, while the expression of GUCA2A did not differ between the groups.*

**Supplementary Information:**

The online version contains supplementary material available at 10.1007/s00431-023-04907-3.

## Introduction

Necrotizing enterocolitis (NEC) is a life-threatening disease that predominantly affects preterm infants. The incidence of NEC has increased [1–6], due to improved survival among extremely preterm infants, consequently increasing the population at risk of NEC [4, 7, 8]. The outcome of NEC can be fatal, with mortality reaching up to 100% in the most severe cases [9].

Since NEC was first described in 1965 [10], attempts have been made to understand the etiology and pathogenesis of the disease [11]. Current knowledge suggests multifactorial causes of NEC [12], including risk factors such as prematurity [13], being small for gestational age [14], and low birth weight [15, 16]. The immature intestinal barrier has been proposed to promote bacterial translocation and infection, followed by an excessive and unregulated inflammatory response in the preterm infant [17]. In combination with a dysfunctional regulation of circulation, this leads to widespread intestinal injury and necrosis [4].

The intestinal barrier function has attracted attention in studies of NEC, specifically the Paneth cells as they play a role in the intestinal immune system, regulate intestinal blood flow, respond with local and systemic inflammation, and express growth factors [18–20]. Paneth cells have also been linked to inflammatory bowel diseases [21, 22]. In animal models of simulated NEC, the involvement of Paneth cells has been established [23, 24], and dysfunctional Paneth cells have recently been linked to the development of NEC in humans [20, 23]. One specific marker for human Paneth cells is defensin alpha 6 (DEFA6) [25]. As results from studies of alpha-defensin expressions in NEC [26–28] have hitherto been inconsistent, we included a second protein marker in order to strengthen our results on Paneth cells in the developing bowel and the possible association with NEC development. A survey of the Human Protein Atlas showed that 93 proteins are associated with Paneth cells, and that guanylate cyclase activator 2A (GUCA2A) is the most specific protein for Paneth cells [29]. Furthermore, GUCA2A was considered to be important for studies of NEC since it has been described to be involved in suppressing intestinal inflammation, [30] to be downregulated in colorectal cancer [31] and inflammatory bowel disease [32, 33], and has to our knowledge not yet been studied in NEC.

The aim of this study was to explore the expression of the Paneth cell markers DEFA6 and GUCA2A in the histologically intact intestine of preterm infants with surgically treated NEC, as possible tissue markers for NEC.

## Methods

### Study setting and participants

Forty-three preterm infants with NEC who underwent intestinal resection and stoma formation at Uppsala University Children´s Hospital, Uppsala, Sweden, between 2003 and 2019 were prospectively recruited for the study (NEC group). Twenty-seven infants that underwent intestinal resection due to intestinal atresia (*n* = 16), dysmotility caused by intestinal immaturity (*n* = 4), aganglionosis (*n* = 4), pseudo-obstruction (*n* = 1), volvulus (*n* = 1), and one (*n* = 1) negative laparotomy served as controls (Controls) were also included prospectively during the same time period. In addition, three adult controls were included as positive controls for orientation of protein expression. All tissue samples in this study were collected from alive individuals in order to avoid post-mortem tissue changes. This study has been approved by the Regional Ethical Review Board, D:no. 2019–00,437. Written informed consent was obtained from the parents of the included infants and from the included adults.

NEC was defined as Bell stage ≥ IIa [34]. The diagnosis was established from clinical and radiographic findings, and was confirmed during surgery and histopathological evaluation. The treatment before surgery consisted of broad-spectrum antibiotics, discontinuation of enteral feeding, and bowel relief. Only samples of histologically intact intestine from the ends of the resected intestine were included in this study. Absence of signs of necrosis, such as nuclear changes and tissue disruption, in the included tissue samples was validated by a pathologist who was blinded for the clinical diagnosis.

### Immunohistochemical analysis of tissues

All tissue samples were sectioned and stained during the same occasion to minimize the risk of lab practice variations that can lead to differences in staining quality. The tissues were fixed in 4% formaldehyde in PBS at 4 °C for 24 h. The samples were sectioned with a width of 3 μm. After being mounted on SuperFrost slides, the samples were de-paraffinized with xylol-ethanol and stained with hematoxylin–eosin. Expression of DEFA6 and GUCA2A was then separately determined immunohistochemically with Benchmark Ultra system (Ventana, Roche Diagnostics, Solna, Sweden) and the ultraView Universal DAB Detection Kit (Ventana) in samples from each individual. The Benchmark Ultra system provides standardized and fully automated immunohistochemistry slide staining, minimizing slide to slide variations in staining intensity and quality. The tissue samples were incubated with a polyclonal rabbit antibody with either anti-DEFA6 or anti-GUCA2A (Prestige Antibodies® Powered by Atlas Antibodies, 1:800 dilution). This was performed after antigen retrieval in CC2 buffer (Ventana) for 36 min. For Controls, tissue sections were prepared without inclusion of primary antibodies. Stained sections were scanned by digital slide scanner NanoZoomer S60 (Hamamatsu, Japan) and with the same exposure times. Digitalized sections were examined by a full slide viewing software (NDP.view2, Hamamatsu). The same magnification (10 × objective) was used for all the images. Each staining was manually controlled to ensure sufficient quality and that the stained tissue samples included histologically intact mucosa. The chosen immunohistochemistry method preserved the possibility of evaluating the structure of the tissue and not only the distribution of the Paneth cell–specific proteins. Three representative areas per section and patient were exported into three images (size: 23 MP, 6400 × 3616 pixels, type: RGB, format: TIFF).

### Image analysis

**Fig. 1:**
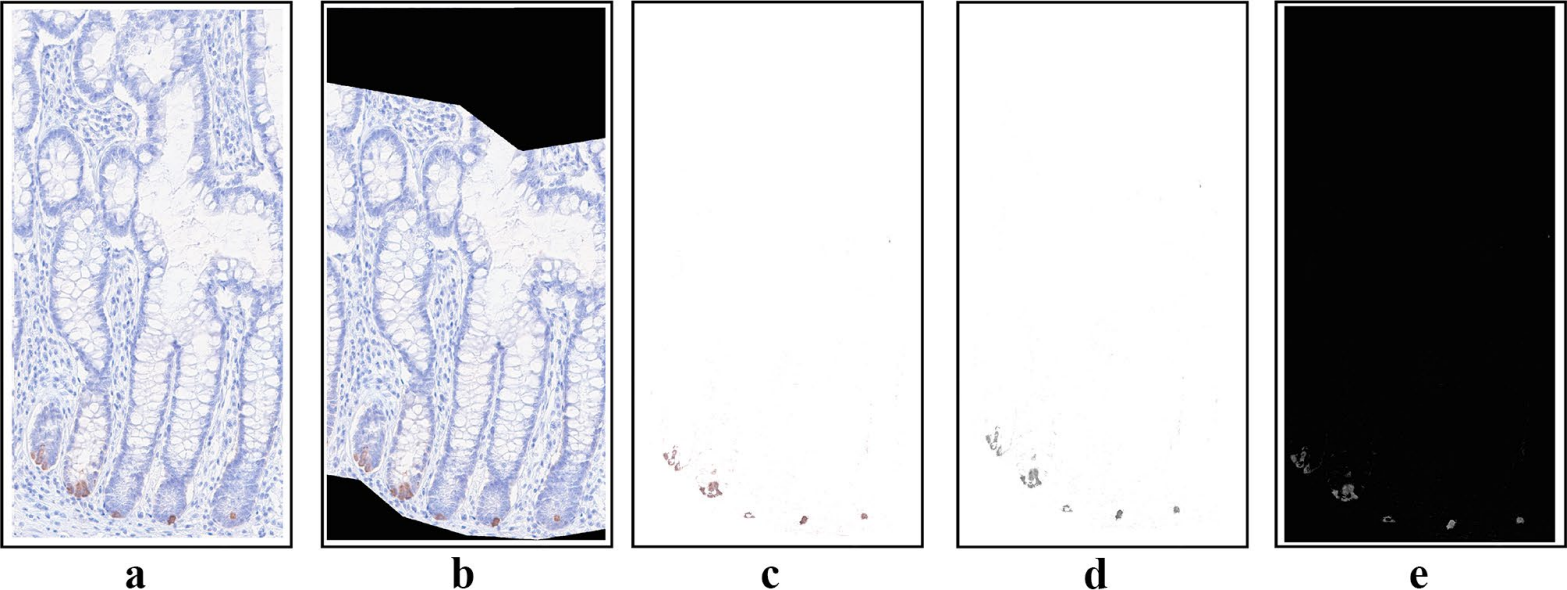
**a**–**e** Image processing to detect DAB staining. **a** Digital image of the immunohistochemically stained tissue sample in RGB color. **b** Selected region of interest (ROI), stretching 500 µm above the basal lamina. **c** Color selection with the IHC Toolbox plugin in ImageJ, representing DAB staining. **d** RGB image converted into an 8-bit image; lowest pixel intensity represents the highest DEFA6 expression and vice versa. **e** Pixel intensity is inverted, so the highest pixel intensity corresponds to the highest DEFA6 expression

A total of 210 images were sorted into stack and saved in TIFF format. Image analysis was executed blindly and performed semi-automatically by ImageJ (freeware, National Institutes of Health [NIH] USA) and the IHC Toolbox plugin [35]. The workflow of image processing is displayed in Fig. 1a–e. A representative area, i.e. region of interest (ROI), of the mucosa was manually chosen from each individual image, stretching from the basal lamina with a standardized height of 500 μm to ensure a consistent section for protein expression comparison (Fig. 1b). In each individual, expression of both DEFA6 and GUCA2A was analyzed. Expression of DEFA6 and GUCA2A was detected with the DAB staining, where the color intensity corresponds to expression level. The model for color detection was adjusted for this project and set to a specific level in DEFA6 and GUCA2A respectively. A threshold window was set to filter unspecific high or low pixel values (Fig. 1c). The accuracy of the model was validated visually. The RGB images of DAB stained images were converted to 8-bit files (Fig. 1d) and inverted (Fig. 1e), so a higher pixel intensity corresponds to a higher protein expression. In each individual, three separate representative ROIs were located and analyzed to give a more nuanced and representative description of the biomarker expression in the given individual. An average protein expression was calculated from these three ROIs.

### Analysis of protein expression

**Fig. 2:**
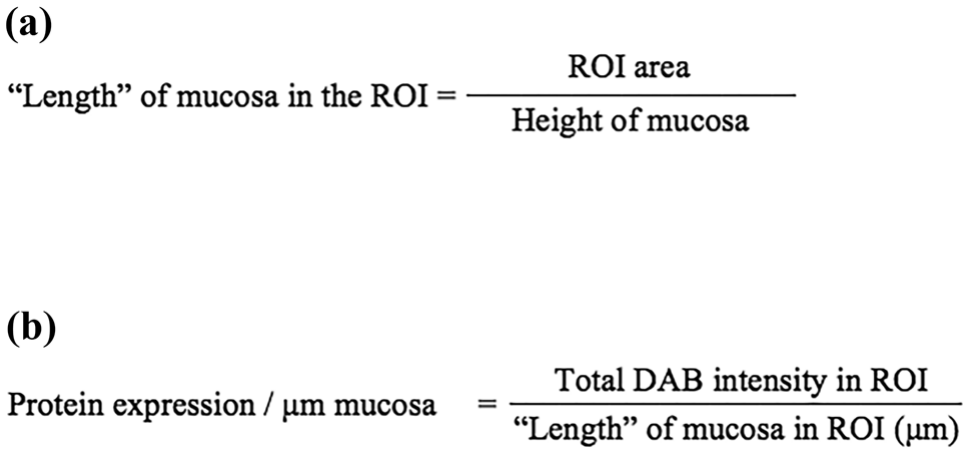
Equations used to define protein expression in each tissue sample. **a** The region of interest area (ROI) was divided by the height of mucosa (500 µm). This gives the “length” of mucosa in the selected ROI in the tissue sample. **b** The DAB intensity is divided with the “length” of mucosa, found in **a**; the result is protein expression/μm mucosa

A standardized process was generated to enable comparison of protein expression. We hypothesized that the distribution of the protein expression was homogenous and had uniform intensity in all tissue samples. The ROI had the same height in each individual image and had its base in the basal membrane (Fig. 2). Protein expression was measured per μm length of the mucosa according to Eqs. (a) and (b). The ROI area was divided by the height of the mucosa (500 μm) which gave the “length” of the mucosa in the sample (a). The total DAB intensity was divided by the “length” (a), resulting in the protein expression per μm mucosa (b).

### Statistical analysis

A *p*-value < 0.05 was considered statistically significant. All statistical calculations were performed in Microsoft Excel 15.27 (161,010) and SPSS version 26. Two-sided Student’s *t*-test was used for comparison of continuous variables, and chi-square test for categorical data. The Mann–Whitney *U* test was used to compare medians. Logistic regression analysis was performed to see how protein expression influences the risk of developing NEC. Linear regression analysis was performed to see how clinical data co-vary with protein expression.

## Results

### Clinical characteristics

**Fig. 3:**
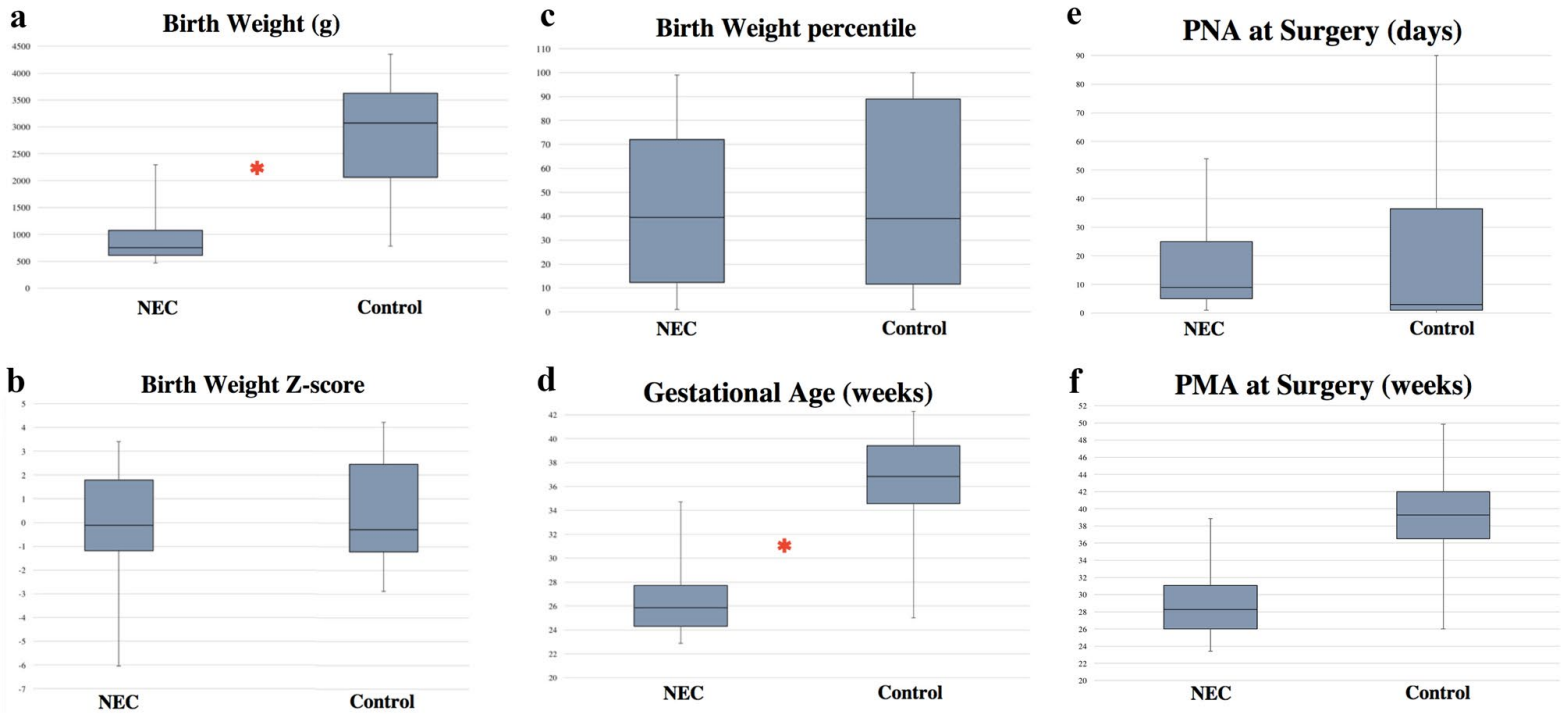
**a**–**f** Clinical data. Statistical difference when comparing the NEC group and Controls is marked with *. **a** Birth weight in grams. **b** *Z*-score for birth weight. **c** Birth weight in percentiles. **d** Gestational age in weeks. **e** Postnatal age (PNA) in days. **f** Postmenstrual age (PMA) in weeks

Clinical characteristics of the NEC group and the Controls can be found in Table 1. The distribution of gender was similar in both groups. The majority of the tissue samples were collected from the ileum (Table 1). Mean *birth weight* was lower in the NEC group compared to the Controls (Table 1 and Fig. 3a). There was no difference between the NEC group and Controls when adjusting the birth weight to *birth weight Z-score* (*p* = 0.220) and *birth weight percentile* (*p* = 0.258) (Table 1 and Fig. 3b–c). The *gestational age* (GA) in both groups ranged from extremely preterm to a maximum GA of 35 weeks in the NEC group and maximum GA of 42 weeks in the Controls (Table 1). The NEC group had a lower mean GA than the Controls (Table 1 and Fig. 3d), and the interquartile range (IQR) of GA did not overlap between the NEC group and the Controls (Table 1). *Postnatal age* (PNA) at surgery did not differ between the two groups (Table 1 and Fig. 3e). The *postmenstrual age* (PMA) at surgery was lower in the NEC group than in the Controls with no overlap in IQR (Table 1 and Fig. 3f). In the NEC group, increased GA did not correlate to decreasing PNA at surgery (*p* = 0.550).

**Table 1:** Clinical and tissue sample characteristics, including age at surgery

	NEC group	Controls	*p*-value
Individuals, *n*	43	27	-
Male, *n* (%) Female, *n* (%)	27 (62.8) 16 (37.2)	15 (55.6) 12 (44.4)	0.762
Location of tissue sample Ventricle, *n* (%) Duodenum, *n* (%) Jejunum, *n* (%) Ileum, *n* (%) Colon, *n* (%) Non-specified intestine, *n* (%)	1 (2.3) 0 (0) 3 (7.0) 27 (62.8) 4 (9.3) 8 (18.6)	0 (0) 0 (0) 4 (14.8) 17 (63.0) 0 (0) 6 (22.2)	- - 0.334 0.994 - 0.090
Birth weight, gram Mean ± SD	910 ± 422.2	2831 ± 1079.3	** < 0.001**
Birth weight, percentile Mean ± SD	41.2 ± 32.6	48.7 ± 38.3	0.458
Birth weight *Z*-score, AU Mean ± SD	− 0.6 ± 1.7	− 0.1 ± 1.7	0.220
Gestational age, weeks Mean ± SD Median (IQR)	26.5 ± 3.0 25.9 (24.3–27.7)	36.1 ± 4.5 36.9 (34.6–39.4)	** < 0.001** ** < 0.001**
Postnatal age at surgery, days Mean ± SD Median (IQR)	15 ± 14.5 9 (5–25)	20 ± 26.8 3 (1–36.5)	0.362 0.128
Post menstrual age at surgery, weeks Mean ± SD Median (IQR)	28.7 ± 3.5 28.3 (26.0–31.1)	39.1 ± 4.6 39.3(36.5–42)	** < 0.001** ** < 0.001**

Statistically significant p-values (*p*<0.05) are found in bold

*n* number, *SD* standard deviation, *AU* arbitrary units, *IQR* interquartile range

### DEFA6

**Fig. 4:**
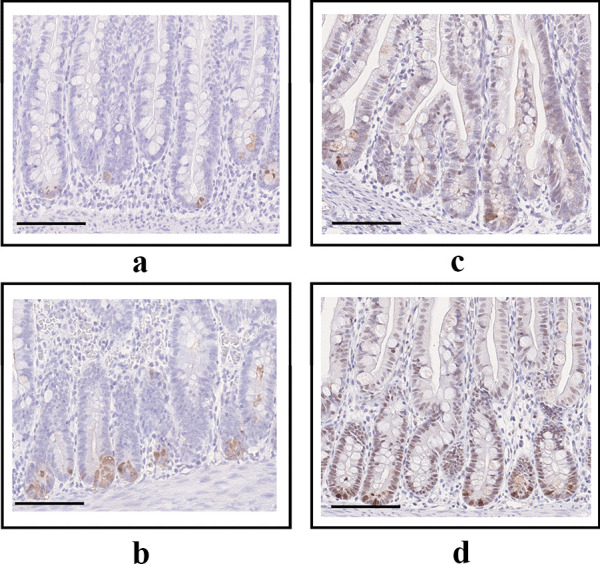
**a**–**d**Representative tissue samples. **a** DEFA6 staining in an individual with NEC. **b** DEFA6 staining in a control. **c** GUCA2A staining in an individual with NEC. **d** GUCA2A staining in a contro

**Fig. 5:**
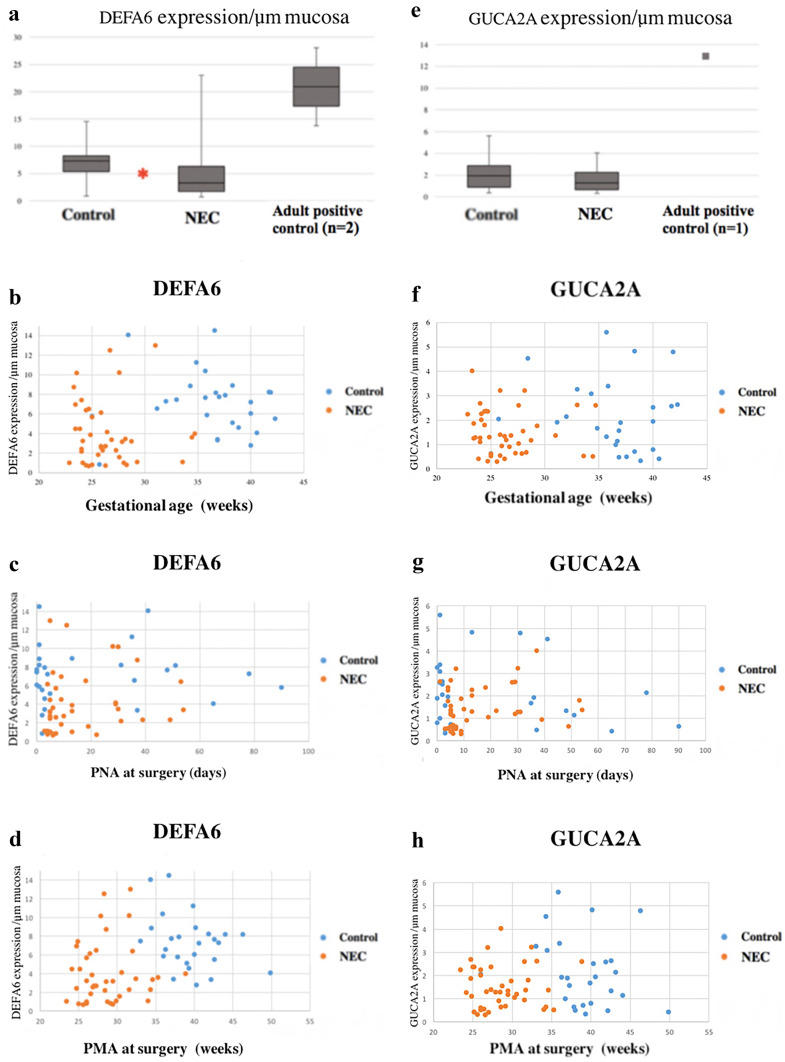
**a**–**h** Protein expression per μm mucosa in NEC group (NEC), Controls, and Adults. **a** Expression of DEFA6 in NEC group, Controls, and Adults. **b**–**d** Scatter plot of DEFA6 correlated to gestational age (**b**), postnatal age (PNA) at surgery (**c**), and postmenstrual age (PMA) at surgery (**d**). * marks significant difference between NEC and Controls. NEC group is marked in orange and Controls in blue. **e** Expression of GUCA2A in NEC group, Controls, and Adult. **f**–**h** Scatter plot of GUCA2A correlated to gestational age (**f**), PNA at surgery (**g**), and PMA at surgery (**h**). NEC group is marked in orange and Controls in blue

Representative stained tissue samples from and individual with NEC and a Control can be found in Fig. 4a and b. Mean DEFA6 expression/μm mucosa was lower in the NEC group (4.56/μm mucosa) compared to the Controls (7.11/μm mucosa) (*p* = 0.006; Fig. 5a). The Adult controls had a mean DEFA6 expression of 20.92/μm mucosa. The NEC group was characterized by a lower GA and a lower expression/µm mucosa of DEFA6 than Controls (*p* < 0.001 and *p* = 0.006 respectively; Fig. 5b). The linear regression analysis to predict DEFA6 expression/μm mucosa based on clinical features displayed significant albeit weak correlations with PMA and PNA (*r* values < 0.300; Table 2).

**Table 2:** Correlation between DEFA6 or GUCA2A and clinical parameters

Dependent variable	Independent variable	*r* value	*p*-value
DEFA6	GA	0.165	0.171
DEFA6	PMA	0.277	**0.020**
DEFA6	PNA	0.275	**0.021**
DEFA6	BW	0.192	0.111
DEFA6	BW *Z*-score	0.070	0.565
DEFA6	BW percentile	0.062	0.611
GUCA2A	GA	0.177	0.144
GUCA2A	PMA	0.146	0.227
GUCA2A	PNA	0.041	0.737
GUCA2A	BW	0.157	0.195
GUCA2A	BW *Z*-score	0.079	0.517
GUCA2A	BW percentile	0.056	0.647

Statistically significant p-values (*p*<0.05) are found in bold

*GA* gestational age, *PMA* postmenstrual age, *PNA* postnatal age, *BW* birth weight

### GUCA2A

Representative stained tissue samples from and individual with NEC and a Control can be found in Fig. 4c and d. Expression of GUCA2A/μm mucosa reached similar levels when comparing the NEC group (1.50/μm mucosa) with the Controls (2.14/μm mucosa) *(p* = 0.053), while the Adult control had a markedly higher expression of GUCA2A (12.95/μm mucosa; Fig. 5e). The linear regression analysis did not detect any significant correlations between GUCA2A expression and GA, PNA, PMA, or BW respectively (Fig. 5f–h; Table 2).

### Predicting risk of NEC

Logistic regression analysis was performed to determine the likelihood of developing NEC as correlated to clinical variables and protein expression. A negative correlation was found between NEC and GA, PMA, BW, and DEFA6 expression/μm mucosa (Table 3). In a multivariate logistic regression analysis with NEC entered as the dependent variable and with DEFA6 expression and GA combined as independent variables, a correlation was found (*p* < 0.001), where both DEFA6 (OR 0.806 [CI 0.0668–0.973]; *p* = 0.024) and GA (OR 0.618 [CI 0.501–0.763]; *p* < 0.001) were separately and independently correlated to NEC. GA was chosen since it so closely correlates to risk of NEC, and since GA differed between the groups.

**Table 3:** Risk analysis for developing NEC

	OR (95%CI)	*p*-value
GA	0.634 (0.526–0.764)	** < 0.001**
PMA	0.576 (0.526–0.764)	** < 0.001**
PNA	0.987 (0.964–1.011)	0.297
BW	0.997 (0.996–0.999)	** < 0.001**
BW percentile	0.994 (0.981–1.008)	0.434
BW *Z*-score	0.829 (0.516–1.122)	0.224
DEFA6	0.843 (0.732–0.971)	**0.018**

Statistically significant p-values (*p*<0.05) are found in bold

*GA* gestational age, *PMA* postmenstrual age, *PNA* postnatal age, *BW* birth weight

### DEFA6 and GUCA2A

No significant relationship was found in the linear regression for DEFA6 expression/μm mucosa in relation to GUCA2A expression/μm mucosa in the NEC group (*p* = 0.425) or in the Controls *(p* = 0.051). In Supplement 1, expression of GUCA2A and DEFA6 is displayed, where the NEC group and the Controls are divided into three gestational groups (Extremely Preterm, Very Preterm, and Mildly Preterm to Term). It shows that both high and low DEFA6 levels occur in the presence of high GUCA2A levels, which confirms that Paneth cells are present and viable even in the presence of low DEFA6 expression.

## Discussion

This study showed that histologically intact intestinal tissue samples from preterm infants with NEC display a diminished expression of DEFA6 along with a maintained GUCA2A expression. These findings were independent of maturation and birth weight in this cohort of preterm infants. DEFA6 expression did not correlate to the expression of GUCA2A, indicating that these protein expressions are independent of each other. The study suggests that NEC patients have well-defined Paneth cells, as shown by preserved GUCA2A expression, and that a dysfunctional defensin activity may be present (Fig. 5).

DEFA6, an alpha-defensin, is a cytotoxic and antimicrobial peptide involved in the innate immune system in the human bowel [25, 36, 37]. DEFA6 is expressed in the secretory granules of Paneth cells in the small intestine and in the colon, and contributes to an antimicrobial shield [25]. Defective production of alpha-defensins has been linked to inflammatory bowel disease [38]. A few studies have investigated alpha-defensin expression (DEFA6 and DEFA5) in NEC [26–28]. The results have been inconsistent, showing that defensin levels may be increased [27, 28] or diminished [26] in NEC. The present study with a relatively large study population found that NEC was linked to diminished DEFA6 expression. DEFA6 is expressed from gestational week 13, but has a low expression until the third trimester is reached [39]. Although this study did find that the expression was lower in preterm infants than in adults, our study did not find that DEFA6 expression correlated to gestational age within this infant population. The study found that susceptibility to NEC increases by lower DEFA6 expression, independently of gestational age.

Guanylate cyclase activator 2A (GUCA2A), a ligand for guanylate cyclase-C [32], is expressed in human Paneth cells [29, 40]. The guanylate cyclase-C (GC-C) signaling pathway is important for the intestinal barrier by regulating tight junctions and intestinal immune system activation against bacterial lipopolysaccharides [41]. Damage of the intestinal barrier and bacterial translocation is considered to be key components in the development of NEC [7]. As GUCA2A has been described to be downregulated in inflammatory bowel disease [32, 33], this study aimed to study GUCA2A and its potential link to NEC, which to our knowledge has not previously been studied. We did not observe that GUCA2A expression was lower in NEC individuals as compared to the Controls. This suggests that NEC individuals do have a normal amount of functioning Paneth cells, and that it is not defective GUCA2A signaling that is involved in NEC development. This warrants further studies of DEFA6, for instance in the form of blood samples taken sequentially.

Postulated theories on the association between Paneth cells and NEC consist of Paneth cells being fewer in number and/or that the Paneth cells are either over-reactive or dysfunctional [42]. Paneth cells initially appear in the first trimester [43] and start to maturate by GA 22–24 weeks [39]. From GA 29 weeks, a significant increase in functioning Paneth cells is found [42] but the amount of cells continues to be lower than in term infants until term gestation is reached [39]. Even though the number of functioning Paneth cells is developmentally regulated, our study could not find a correlation between gestational age and expression of the Paneth cell markers DEFA6 or GUCA2A.

NEC typically appears 1–2 weeks after birth [7]. Thus, the risk of developing NEC to some extent increases with increasing PNA. More preterm infants, however, typically develop NEC later than more mature infants [44]. In our study, PNA did not correlate to risk of NEC. The reason for this could be that our NEC group consists of individuals with varying gestational age, from extremely preterm to mildly preterm.

### Strengths and limitations

This study defines the expression of DEFA6 and GUCA2A in viable tissue before it is histologically affected by NEC, and enables comparison of protein expression in NEC individuals and controls. It was important to only include tissue samples that had not yet been histologically damaged, since non-viable tissue would be difficult to analyze and give inconclusive results. The strength of this study is the large sample size of intestinal tissue from NEC infants and control samples from newborn infants that underwent intestinal resection due to other surgical conditions. Studying Paneth cell development in alive humans is valuable since the developmental expression of DEFA6 may differ from other species resulting in difficulties to translate fully findings from animals to humans [39]. The majority of tissue samples were collected from the ileum, which is the location with most Paneth cells, followed by the colon [45], which are the most common sites to be affected by NEC [46]. The study included infants with progressed NEC, i.e., NEC stages where surgery was needed, and thus our findings can only be considered applicable in late stages of NEC.

When using image analysis of stained tissue samples for quantification of a biomarker, it is important to mitigate risks of analyzing non-representative biomarker detections. This was done through standardization of lab practice, manual control of the quality of the stain, and by calculating a mean biomarker level from three separate representative ROIs from each individual. Furthermore, the surgical and histopathological confirmation of the NEC diagnosis ensures that these findings are specific for NEC.

A potential obstacle when analyzing the presence of Paneth cells and their involvement in NEC is that a detected low protein expression could indicate other aspects of their cell function. It could represent a low number of Paneth cells, or that the Paneth cells are unable to synthesize and excrete the protein [20]. The simultaneous analysis of two selective Paneth cell markers in our study reveals that functioning Paneth cells are present in NEC individuals, and that the DEFA6 expression is diminished.

A limitation was that the controls were not completely healthy. However, collecting tissue samples from completely healthy controls was not feasible due to obvious ethical reasons. Aganglionosis has been linked to Paneth cell hyperplasia and metaplasia in murine models [47] which may influence Paneth cell marker expression in our controls. Another limitation was that the infants with NEC had a lower GA, birth weight, and PMA than the Controls. The individuals in the NEC group had a relatively low gestational age. As previously reported, our unit has a long history of caring for extremely preterm infants born at very low gestational ages and with a high survival rate, which has resulted in a lower postnatal age at start of developing NEC, which has also been reported from other centers [3, 48]. It is difficult in this kind of study to find matched live Controls, not post-mortem samples, since the timing of disease onset and the clinical features of the affected individual can vary greatly. To compensate for the difference in clinical data, statistical calculations were used. The difference in birth weight was not present when adjusted to *Z*-score and birth weight percentile, indicating that both groups were equally mature for gestational age. These clinical factors could influence protein expression patterns in the respective groups studied and the risk of developing NEC. The regression analysis was performed to see if protein expression varies with clinical factors. Since the control samples were gathered from individuals who already required surgery, it was not possible to choose paired controls that adequately could have correlated to the clinical features of the NEC group. Nevertheless, the regression analyses show that the significant difference in DEFA6 expression between the groups was independent of gestational age and birth weight.

## Conclusion

Our results suggest that there seems to exist a correlation between defective Paneth cell activity and the development of NEC. Histologically, intact Paneth cells are present in infants with NEC as in the Controls, indicated by the same expression of GUCA2A in both groups. The infants affected by NEC, however, had a lower expression of DEFA6. These observations of GUCA2A and DEFA6 expression were independent of GA and birth weight. Hence, defective postnatal expression of DEFA6 could make infants more prone to develop NEC, an observation that warrants further investigation in future studies.

## Supplementary Information

Below is the link to the electronic supplementary material.

Supplementary file1 Depiction of GUCA2A and DEFA6 expression in different gestational groups. The NEC-group and the Controls are both divided into three gestational groups (Extremely Preterm, Very Preterm and Mildly Preterm to Term)(DOC 330 KB)

## Data Availability

All data supporting the findings in this study can be provided if requested.
