# Supplementary figures and images for: Paneth cell proteins DEFA6 and GUCA2A as tissue markers in necrotizing enterocolitis
Source: Eur J Pediatr. 2023 Apr 5;182(6):2775–84. doi: 10.1007/s00431-023-04907-3 (PMC10257617; doi:10.1007/s00431-023-04907-3)

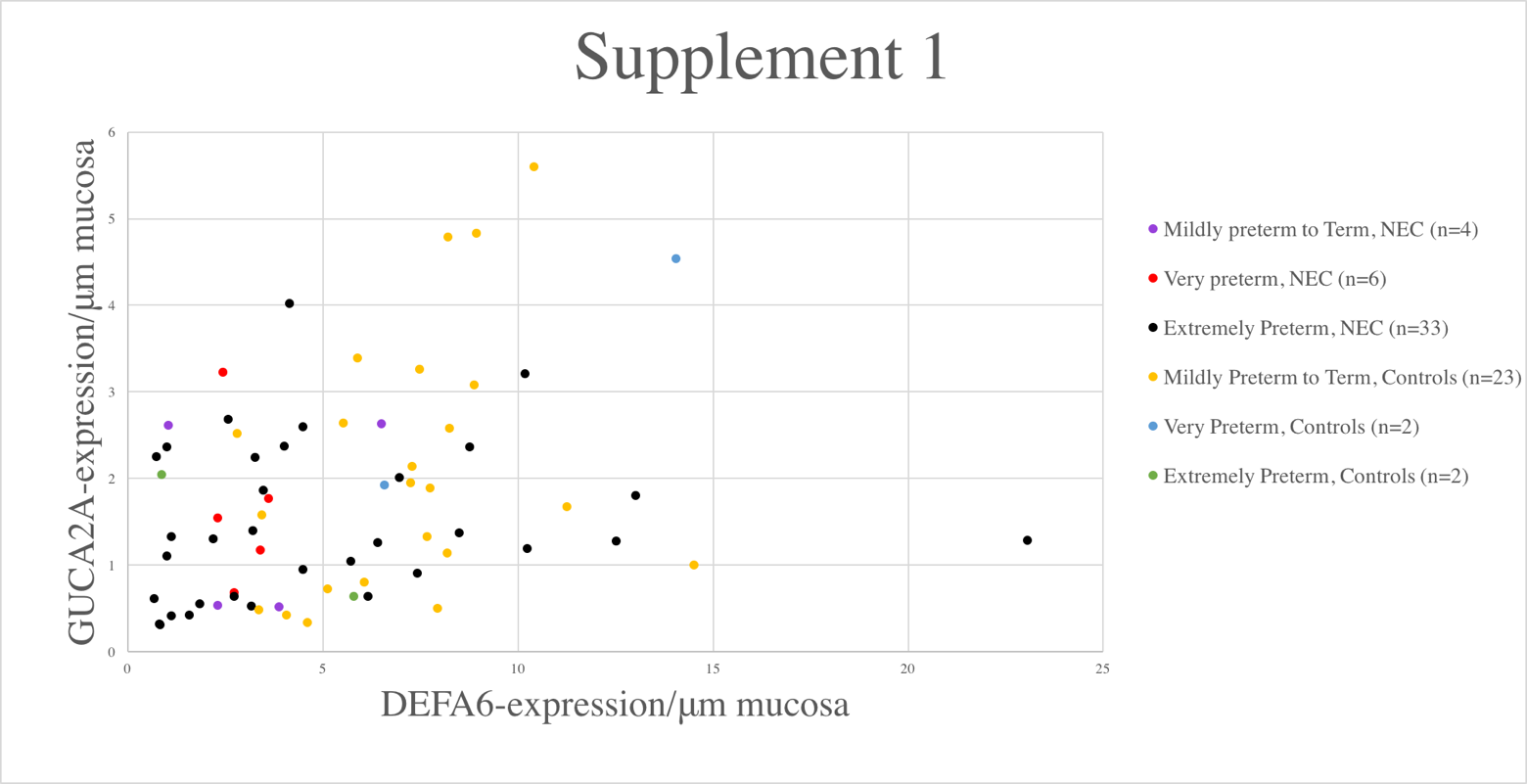

Supplement: Supplementary file 1 — Supplementary file1 Depiction of GUCA2A and DEFA6 expression in different gestational groups. The NEC-group and the Controls are both divided into three gestational groups (Extremely Preterm, Very Preterm and Mildly Preterm to Term)(DOC 330 KB) [file 431_2023_4907_MOESM1_ESM.doc]
